# Beta-Hydroxybutyrate Enhances BDNF Expression by Increasing H3K4me3 and Decreasing H2AK119ub in Hippocampal Neurons

**DOI:** 10.3389/fnins.2020.591177

**Published:** 2020-10-28

**Authors:** Erling Hu, Huan Du, Sen Shang, Yali Zhang, Xiaoyun Lu

**Affiliations:** Key Laboratory of Biomedical Information Engineering of Ministry of Education, School of Life Sciences and Technology, Xi’an Jiaotong University, Xi’an, China

**Keywords:** beta-hydroxybutyrate, calcium, CaMKII, H3K4 tri-methylation, H2AK119 mono-ubiquitination

## Abstract

Neurological evidence suggests that beta-hydroxybutyrate (BHBA) has positive effects on the central nervous system. Previous studies have explored the molecular mechanisms by which BHBA affects different brain functions, but the effects of BHBA on epigenetic modifications remain elusive. Here, we showed that BHBA enhanced brain-derived neurotrophic factor (BDNF) expression by increasing H3K4me3 and decreasing H2AK119ub occupancy at the *Bdnf* promoters I, II, IV, and VI in hippocampal neurons. Moreover, BHBA treatment induced the upregulation of H3K4me3 and downregulation of H2AK119ub on the global level, both of which were dependent on the L-type calcium channel. Additionally, the BHBA-activated L-type calcium channel subsequently triggered the activation of Ca^2+^/CaMKII/CREB signaling, and promoted the binding of p-CREB and CBP to *Bdnf* promoters. These results indicate that BHBA regulates cellular signaling and multiple histone modifications to cooperatively modulate BDNF, suggesting a wide range of regulatory effects of BHBA in the central nervous system.

## Introduction

Beta-hydroxybutyrate (BHBA) has recently gained increasing attention due to its beneficial effects, such as eliciting advantageous changes in metabolism, reducing inflammation and oxidative stress, as well as improving cognition (Tieu et al., 2003; Reger et al., 2004; Shimazu et al., 2013; Fu et al., 2015). The protective effects of BHBA in the brain have been widely studied. BHBA levels in the body are elevated on a ketogenic diet, during caloric restriction or physical excise and either of these states was found to alleviate some pathological symptoms of neurodegenerative diseases such as Alzheimer’s disease, Parkinson’s disease, epilepsy, and ischemia (Suzuki et al., 2002; Tieu et al., 2003; Lutas and Yellen, 2013; Yin et al., 2016). Furthermore, BHBA also exerts beneficial effects under normal physiological circumstances, for example by enhancing the learning and memory ability of healthy mice (Zou et al., 2009). Exercise-induced BHBA elevation promotes the expression of brain-derived neurotrophic factor (BDNF) in the hippocampus (Sleiman et al., 2016). However, although many studies explored the underlying mechanisms, they are still not fully understood. In addition to serving as an endogenous energy source that can be used to synthesize ATP in the brain, BHBA is increasingly understood to have broad cellular signaling functions. These include inhibiting class I histone deacetylases (Shimazu et al., 2013), binding to G-protein-coupled receptors (GPR) (Newman and Verdin, 2017), inhibiting NLRP3 activation (Youm et al., 2015), preventing autophagosome accumulation (Camberos-Luna et al., 2016), repressing H3K27me3 levels (Hu et al., 2018), and acting as a substrate for posttranslational modification of proteins via beta-hydroxybutyrylation at lysine sites (Xie et al., 2016).

Many studies have revealed that BHBA induces BDNF expression when the supply of glucose is insufficient. Our previous study also showed that BHBA was able to promote BDNF expression under normal energy supply (Hu et al., 2018). BDNF is an important trophic factor associated with the improvement of brain function and significant neuroprotective effects. BDNF is a small dimer synthesized and widely distributed in different brain regions, including the hippocampus, amygdala, cortex, forebrain, striatum, and hypothalamus (Aid et al., 2007). In general, the expression of BDNF is controlled by multi-component transcriptional regulation including cAMP response element (CRE)-binding protein (CREB) and epigenetic factors such as histone modifications. The transcriptional activation marks histone H3 lysine 4 trimethylation (H3K4me3) and histone H3 lysine 27 acetylation (H3K27ac), as well as the transcriptional repression mark H3K27 trimethylation (H3K27me3), can directly regulate *Bdnf* transcription in active or repressive ways, leading to changes of promoter occupancy and BDNF expression (Bernstein et al., 2006; Palomer et al., 2016; Hu et al., 2018). The *Bdnf* gene contains nine exons, each of which is regulated by its promoter. Generally, the expression of transcripts from *Bdnf* exons I, II, IV, and VI increases with time, and is important for brain development (Zheng et al., 2012). Interestingly, H3K4me3 and H3K27me3 are consistently distributed in *Bdnf* promoters I to VIII in a strongly bivalent way, so that the gene can be turned on or off rapidly in response to environmental stimuli (Bernstein et al., 2006). In addition, other histone marks, including histone H2A lysine 119 mono-ubiquitination (H2AK119ub) that play a vital role in transcriptional repression, can also co-localize with H3K27me3 in the same chromatin regions to regulate gene transcription (Muller and Verrijzer, 2009). BHBA can increase BDNF expression by elevating the occupancy of H3K27ac at the *Bdnf* promoters and decreasing that of H3K27me3 (Palomer et al., 2016; Hu et al., 2018), suggesting a yet to be investigated potential role of BHBA in regulating histone modifications. However, the locational interaction between the different histone modifications, for example, H3K27me3 and H3K4me3, indicates that the function of BHBA might not just be limited to affecting H3K27me3/ac, but also the other histone marks.

Here, we demonstrated that BHBA enhanced BDNF expression by elevating the transcriptional activation mark H3K4me3 and decreasing the transcriptional repression mark H2AK119ub in hippocampal neurons both *in vivo* and *in vitro*. Furthermore, both the BHBA-triggered increase of H3K4me3 and the decrease of H2AK119ub depend on the L-type calcium channel. These results corroborate the broad functions and diverse mechanisms by which BHBA regulates neuronal genes. The possibility that BHBA modulates the expression of additional neuronal genes to achieve its globally beneficial effects merits further investigation in future studies.

## Materials and Methods

### Animals

The *in vivo* experiments were conducted with 6- to 8-week-old female C57BL/6 mice. The mice were maintained as described previously (Hu et al., 2018). The mice were treated with 60 mg/kg BHBA (dissolved in physiological saline) or only physiological saline twice daily via intragastric gavage for two days in total. The hippocampi were removed and stored at −80°C before further analysis. For *in vitro* experiments, rat hippocampi were isolated from 1-day-old SD rats for culturing primary hippocampal neurons. All experimental procedures involving animals were in strict accordance with institutional, national, and international animal welfare standards, and were approved by the Research Ethics Committee of Xi’an Jiaotong University (Approval No. 201103). All animals were purchased from the Experimental Animal Center of Xi’an Jiaotong University Health Science Center. All mice were fed a normal diet at libitum and were maintained on a 12-h light/dark cycle.

### Culture of Primary Hippocampal Neurons

Primary hippocampal neurons from 1-day-old SD rats were extracted and cultivated as described previously (Hu et al., 2018). The hippocampi were isolated in ice-cold Hank’s solution and digested with 0.25% trypsin at 37°C for 15 min, followed by centrifugation for 5 min at 300 × *g*. The pellets were re-suspended in Dulbecco’s Modified Eagle’s Medium (DMEM)/F12 medium (high glucose; hyclone, United States) with 10% fetal bovine serum (FBS, HyClone, GE Healthcare, United States), supplemented with 100 U/ml penicillin (Beyotime, China) and 100 μg/ml streptomycin (Beyotime, China). The cells were counted and then seeded into plates pre-coated with poly-L-lysine (Sigma-Aldrich, United States). After 2 h, the medium was replaced with Neurobasal^TM^ A Medium supplemented with 2% B27 and 2 mM Gln. Half of the medium was changed every 3 days.

### Culture of Murine Hippocampal Neuronal Cell Line HT22

The HT22 cell line was purchased from Shanghai Hongshun Biologicals (China) and maintained in high-glucose DMEM supplement with 10% FBS, 100 U/ml penicillin, and 100 μg/ml streptomycin.

### Western Blot Analysis

Rat hippocampal neurons were cultured in 6-well plates at an initial concentration of 2 × 10^6^ cells per well. After culturing for 9–10 days, the neurons were treated with 10 μM manidipine, 5 μM KN-62, or 10 μM H89 for 10 min, or left untreated prior to treatment with 2 mM BHBA for another 1 or 12 h. Subsequently, the cells were lysed using 1× lysis buffer (Beyotime, China) containing protease inhibitor cocktail 2 (Roche, Switzerland) directly in the plates to obtain whole-cell lysates. Proteins were separated on SDS/PAGE gels and electroblotted onto PVDF membranes (GE Healthcare, United States). The membranes were blocked with 5% (w/v) BSA (bovine serum albumin; Sigma-Aldrich, United Kingdom) for 1 h at room temperature, and subsequently incubated with primary antibodies (tri-methyl histone H3 (Lys4) rabbit mAb, ubiquityl-histone H2A (Lys119) rabbit mAb, phospho-CaMKII (Thr286) rabbit mAb, phospho-CREB (SER133) rabbit mAb, WDR5(A-6), anti-BDNF antibody, anti-beta-actin) at 4°C overnight. Then, TBST was used to wash the membranes 3 times, followed by incubation with the horseradish peroxidase (HRP)-conjugated secondary antibody (Abcam, United Kingdom) for 1 h at room temperature. The protein bands were developed using the ImmobilonTM Western Chemiluminescent HRP Substrate (Millipore, United States) and photographed using the FlorChem FC2 System (Alpha Innotech, United States).

### Chromatin Immunoprecipitation Followed by Quantitative PCR

Chromatin immunoprecipitation (ChIP) assays were processed using the SimpleChIP Enzymatic Chromatin IP kit (Catalog#9003, Cell signaling Technology) according to the manufacturer’s instructions. Briefly, HT22 cells were fixed in 1% formaldehyde for 10 min at room temperature, after which 0.125 M glycine was used to stop the cross-linking for 5 min at room temperature. Then, the samples were washed 2 times with ice-cold PBS supplemented with 1× protease inhibitor cocktail (PIC; Roche, Switzerland). Subsequently, ice-cold 1× Buffer A plus PIC and DTT were added and the cells were incubated on ice for 10 min. The resulting lysates were centrifuged at 2,000 × *g* for 5 min at 4°C to harvest the pellets. Subsequently, 1× Buffer B plus DTT was added to lyse the nuclei, followed by 0.5 μl micrococcal nuclease per IP prep and incubated at 37°C to digest DNA to a length of approximately 150–900 bp. After 20 min, the digestion was stopped by adding 2 μl 0.5 M EDTA. The resulting lysates were centrifuged at 16,000 × *g* and 4°C to harvest the pelleted nuclei. The samples were resuspended in 1× ChIP Buffer with PIC, incubated on ice for 10 min, and then sonicated using a Bandelin ultrasonicator (Sonics, United States) set to 300 W, 50 cycles (1 s each with a duty cycle of 50%). The resulting sonicated mixture was centrifuged at 9,400 × *g* for 10 min at 4°C to remove insoluble material. Supernatants containing DNA-protein complexes were diluted 1:5 with 1× ChIP Buffer. Subsequently, ChIP assays were performed using anti-p-CREB (cell signaling technology), anti-CBP (cell signaling technology), anti-H3K4me3 (cell signaling technology), or anti-H2AK119ub antibodies (Abcam, United Kingdom) and a negative control comprising normal rabbit IgG (Santa Cruz Biotechnology, Inc., United States). Immune complexes were precipitated by adding the magnetic protein G beads and incubating for 2 h at 4°C. The protein G –antibody/chromatin complexes were washed with low salt wash buffer (three times) and a high salt wash buffer (one time). Protein G–antibody/chromatin complexes, as well as the 2% input solution, were eluted with 150 μl 1× ChIP elution buffer two times, and recovered by adding 5 M NaCl and 100 μg/ml protease K and incubating 2 h at 65°C. The beads were separated from the solution using a magnetic separation device and the supernatant was transferred to a fresh tube. Finally, DNA was purified using a DNA purification Kit (DP214; Tiangen, China) according to the manufacturer’s protocol, and eluted with 50 μl sterilized deionized water. A 2 μl aliquot of purified DNA was used to perform qRT-PCR, using the primer pairs *Bdnf*-P1, *Bdnf*-P2, *Bdnf*-P3, and *Bdnf*-P4. Quantitative PCR (qPCR) for the measurement of gene expression was performed using the SYBR Green Master Mix on a CFX96TM Real-time system (Bio-Rad, United States).

### Calcium Imaging

Rat hippocampal neurons cultured for 8 to 10 days were used for the test. The cells were washed with standard 2 mM Hank’s Balanced Salt Solution (HBSS; ThermoFisher Scientific, United States) two times, after which 5 μM Fluo-4AM was used to stain the cells for 15 min, which were then washed again with standard 2 mM extracellular solution. Time-lapse imaging was used at 500 ms/frame for real-time imaging. Neurons were initially given 30 s 2Ca (2 mM Ca^2+^) to record the basal line followed by 1 min of 2 mM BHBA stimulation, and then rapidly switched back to 2Ca. The intensity of Fluo-4AM fluorescence was analyzed using ImageJ Software.

### Enzyme-Linked Immunosorbent Assay

HT22 cells were treated with 2 mM BHBA for 5, 10, or 20 min, or left untreated, and were collected using 0.25% trypsin followed by centrifugation for 5 min at 300 × *g*. The tubes containing the samples were placed into liquid nitrogen for 20 min, and then transferred into a 37°C water bath for 10 min, which was repeated two times. Subsequently, the samples were centrifuged for 5 min at 12,000 *g*, and the supernatants were collected for the detection of PLC activity, using the mouse PLC activity detection kit (Jianglai Biotechnology Company, Shanghai, China) according to the manufacturer’s instructions. Samples comprising 50 μl were added into the wells, after which 100 μl of diluted HRP conjugate was added into each well, and the plate was incubated at 37°C for 60 min. The wells were washed with wash buffer five times, after which 50 μl of chromogenic substrate A and 50 μl of chromogenic substrate B were added into each well, and the plate was incubated in the dark at 37°C for 15 min. Finally, 100 μl of stop solution was added into each well and the absorbance at 450 nm was measured.

### Statistical Analysis

The data were analyzed using GraphPad Prism V7.0 (GraphPad 264 Software, Inc., San Diego, CA, United States). Two-group comparisons were performed using Student’s *t*-test. Multi-group comparisons were performed using a one-way ANOVA followed by *post hoc* Tukey’s test. The error bars indicate the SEM. Differences were considered to be statistically significant at *p* < 0.05.

## Results

### BHBA Treatment Promotes BDNF Expression and Changes Histone H3K4 Trimethylation and Histone H2AK119 Mono-Ubiquitination in *Bdnf* Promoters

**FIGURE 1:**
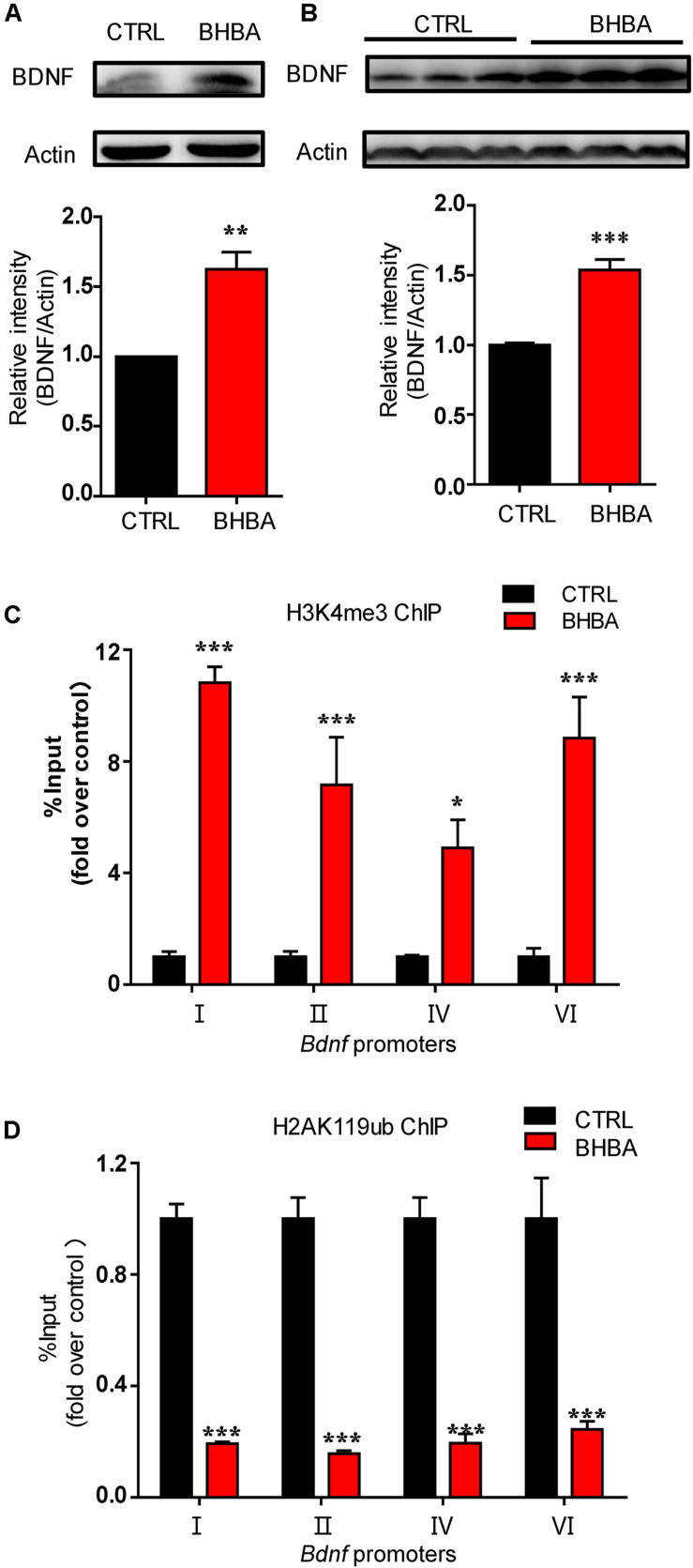
BHBA treatment increased BDNF expression and changed the occupancy of H3K4me3 and H2AK119ub at *Bdnf* promoters in neurons. **(A,B)** Western blot analysis of BDNF levels in hippocampal neurons treated with 2 mM BHBA for 12 h or left untreated (*n* = 4, *F*(3, 3) = 27.46) **(A)**, and in hippocampi of mice treated with 60 mg/kg BHBA, twice 1 day for 2 days and untreated control mice (*n* = 8, *F*(3, 28) = 13.19) **(B)**. **(C,D)** Occupancy of H3K4me3 (*n* = 4, *F*(3, 16) = 0.1304) **(C)** and H2AK119ub (*n* = 4, *F*(3, 16) = 3.828) **(D)** at *Bdnf* promoters from cultured HT22 neurons treated with 2 mM BHBA for 1 h or left untreated. The data represent the mean ± SEM from three independent experiments. *p < 0.05, **p < 0.01, and ***p < 0.001.

Firstly, we verified the increase of BDNF levels in primary hippocampal neurons following treatment with 2 mM BHBA (Figure 1A). Actually, we have previously found that BHBA also could increase BDNF expression in murine hippocampal neuronal cell line HT22 cells (Hu et al., 2018). Additionally, mice were gavaged with 60 mg/kg BHBA, twice a day for two days to further assess the *in vivo* effects of BHBA on BDNF. The BDNF levels in the hippocampi of BHBA-treated mice were much higher than in those of control mice (Figure 1B). Histone modifications play essential roles in chromatin remodeling and transcriptional regulation. Previous studies indicated that BHBA regulates the lysine 27 tri-methylation of histone H3 (H3K27me3) in *Bdnf* promoters (Palomer et al., 2016). The transcriptional activation mark H3K4me3 is found in a constant balance with the permissive H3K27me3 (Bernstein et al., 2006). Firstly, murine hippocampal neuronal cell line HT22 was treated with 2 mM BHBA for 1 h, followed by ChIP with an antibody against H3K4me3 antibody and measurement of *Bdnf* transcription using real-time qPCR. The occupancy of H3K4me3 in *Bdnf* promoters I, II, III, and IV were significantly increased by BHBA stimulation (Figure 1C). It is believed that BHBA broadly regulates epigenetic modification in multiple ways. In addition to H3K4me3, the transcriptional-repression mark histone H2AK119 mono-ubiquitination (H2AK119ub) is also involved in transcriptional regulation, always occurring together with H3K27me3 in the same chromatin regions (Wang et al., 2004). H2AK119ub was found to be directly bound at *Bdnf* promoters I to IV, while BHBA treatment weakened the occupancy of H2AK119ub at these promoters (Figure 1D).

### BHBA Treatment Causes an Increase of H3K4me3 and a Decrease of H2AK119ub in Hippocampal Neurons *in vitro* and *in vivo*

**FIGURE 2:**
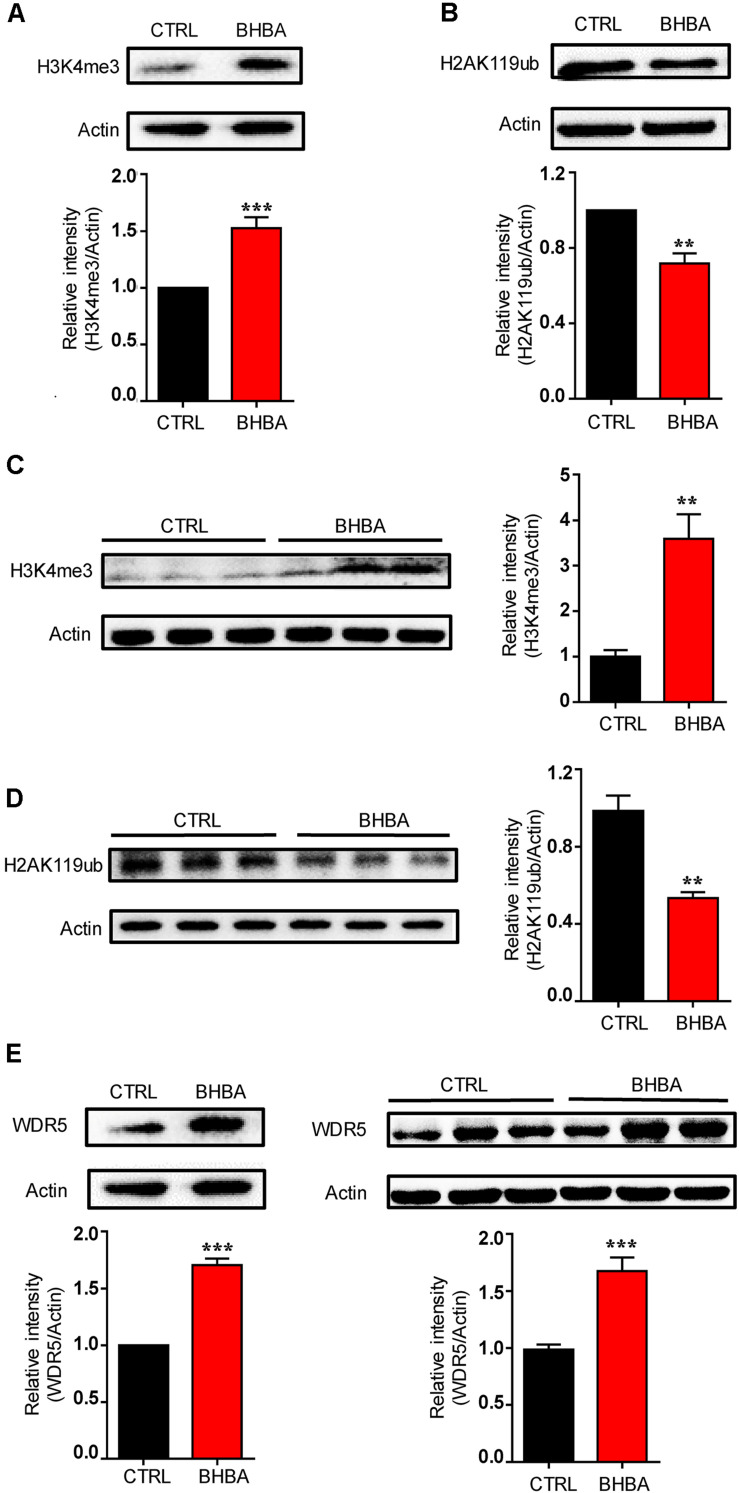
BHBA treatment increased H3K4me3 and decreased H2AK119ub levels in hippocampal neurons both *in vitro* and *in vivo*. **(A,C)** Western blot analysis of H3K4me3 levels in hippocampal neurons treated with 2 mM BHBA for 1 h or left untreated (*n* = 5, *F*(4, 4) = infinity) **(A)**, and in hippocampi of mice treated with 60 mg/kg BHBA, twice one day for two days, or untreated control mice (*n* = 4, *F*(3, 3) = 23.12) **(C)**. **(B,D)** Western blot analysis of H2AK119ub levels in hippocampal neurons treated with 2 mM BHBA for 1 h or left untreated (*n* = 4, *F*(3, 3) = infinity) **(B)**, and in hippocampi of mice treated with 60 mg/kg BHBA, twice 1 day for 2 days, or untreated control mice (*n* = 4, *F*(3, 3) = 7.503) **(D)**. **(E,F)** Western blot analysis of WDR5 levels in hippocampal neurons treated with 2 mM BHBA for 1 h or left untreated (*n* = 3, *F*(2, 2) = infinity) **(E)**, and from hippocampi of mice treated with 60 mg/kg BHBA, twice 1 day for 2 days, or untreated control mice (*n* = 4, *F*(3, 3) = 14.05) **(F)**. The data are presented as mean ± SEM. **p < 0.01 and ***p < 0.001.

The effects of BHBA on the global levels of H3K4me3 and H2AK119ub in hippocampal neurons were investigated. After 1 h of treatment, 2 mM BHBA elevated the global H3K4me3 levels in the primary hippocampal neurons (Figure 2A). Accordingly, the untreated hippocampal neurons exhibited high levels of H2AK119ub, whereas BHBA treatment significantly reduced the global abundance of H2AK119ub (Figure 2B). The *in vivo* results from showed that the total H3K4me3 levels were much higher, and those of H2AK119ub were much lower in the hippocampi of BHBA-treated mice as than those of the control mice, which was consistent with the *in vitro* results (Figures 2C,D).

At the same time, WD repeat-containing protein 5 (WDR5), a core subunit of the mixed-lineage leukemia/SET-domain (MLL/SET) complex, which plays a crucial role in regulating global H3K4me3 levels during vertebrate development and hematopoiesis, was detected both *in vitro* and *in vivo* (Wysocka et al., 2005). WDR5 levels were elevated by BHBA both in cultured hippocampal neurons and hippocampi of mice (Figures 2E,F). These results suggest that BHBA positively regulates H3K4me3, possibly by increasing WDR5 levels in hippocampal neurons. Taken together, these results confirmed the epigenetic regulation of *Bdnf* promoters on H3K4me3 and H2AK119ub in response to BHBA. Therefore, H3K4me3 and H2AK119ub might be involved in regulating BHBA-induced BDNF expression in hippocampal neurons.

### BHBA Treatment Activates Calcium Signaling in Neurons

**FIGURE 3:**
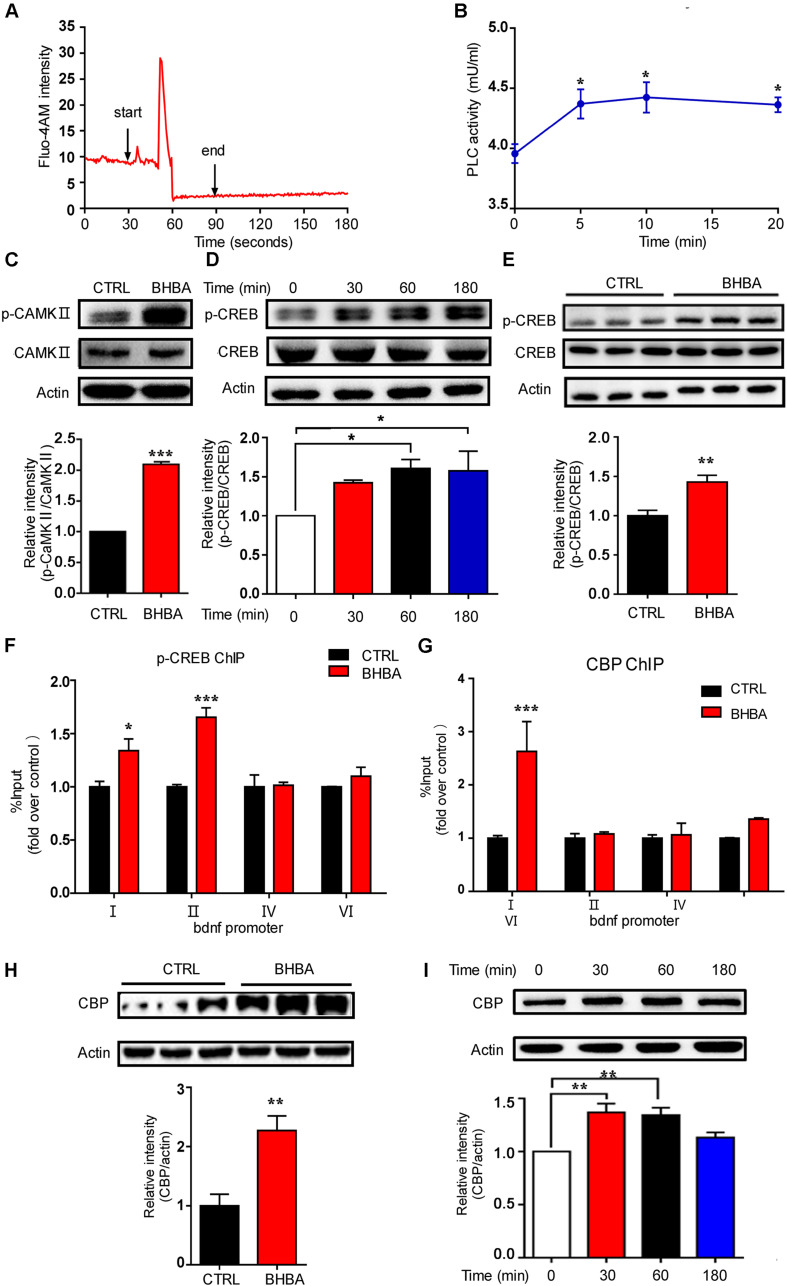
BHBA treatment activated calcium signaling in neurons. **(A)** Representative traces of fluorescence intensity over 3 min of imaging representing intracellular Ca^2+^ concentrations in rat hippocampal neurons stained with Fluo4AM and treated with 2 mM BHBA at the time point of 30 s for another 1 min. **(B)** The activity of phospholipase C (PLC) in rat hippocampal neurons treated with 2 mM BHBA for the indicated time or left untreated (*n* = 3, *F*(3, 8) = 4.589). **(C)** Western blot analysis of p-CAMKII levels from hippocampal neurons treated with 2 mM BHBA for 1 h or left untreated (*n* = 3, *F*(2, 2) = infinity). **(D,E)** Western blot analysis of p-CREB levels in hippocampal neurons treated with 2 mM BHBA for the indicated time or left untreated (*n* = 3, *F*(3, 8) = 3.915) **(D)**, and from hippocampi of mice treated with 60 mg/kg BHBA, twice one day for two days, or untreated control mice (*n* = 4, *F*(3, 3) = 1.583) **(E)**. **(F,G)** Occupancy of p-CREB (*n* = 4, *F*(3, 16) = 7.481) **(F)** and CBP (*n* = 4, *F*(3, 16) = 5.895) **(G)** at the *Bdnf* promoters of HT22 cells treated with 2 mM BHBA for 1 h or left untreated. **(H)** CBP levels in hippocampi of mice treated with 60 mg/kg BHBA, twice one day for two days, or untreated control mice (*n* = 4, *F*(3, 3) = 1.642). **(I)** Western blot analysis of CBP levels in hippocampal neurons treated with 2 mM BHBA for the indicated time or left untreated (*n* = 3 or 4, *F*(3, 11) = 8.651). The data are presented as mean ± SEM. *p < 0.05, **p < 0.01, and ***p < 0.001.

It is believed that changes in epigenetic modifications in cells must be the result of the communication of each cell with the environment, which involves multiple signaling pathways. Calcium signals play crucial roles in these processes. To assess the effects of BHBA on intracellular Ca^2+^ levels, hippocampal neurons were loaded with the calcium-sensitive fluorescence indicator Fluo-4AM, and the changes in fluorescence intensity were measured in all cell bodies of neurons (Figure 3A). The hippocampal neurons were kept in PSS (2 mM Ca^2+^), and PSS was applied for 30 s to obtain the basal Ca^2+^ level. Subsequently, the stimulation was switching from PSS to 2 mM BHBA at the time point of 30 s and maintained for 1 min until the time point of 90 s to switch to PSS again. The intracellular calcium concentration transiently rose after BHBA treatment and was quickly restored to the basal level. Interestingly, another significant rise in intracellular calcium was seen several seconds later (Figure 3A). Moreover, phospholipase C (PLC) activity in the hippocampal neurons was also elevated by BHBA (Figure 3B). The elevation of cellular Ca^2+^ activates several Ca^2+^-binding proteins, including calmodulin (CaM) complexes, and promotes the phosphorylation of CaMKII at a threonine residue (T286) in the autoinhibitory domain. In this study, the phosphorylation levels of (CaMKII) in the hippocampal neurons treated with BHBA for 30 min were much higher than in the controls (Figure 3C). Significant upregulation of phosphorylated CREB (p-CREB), which is an essential component downstream of p-CaMKII, was also seen in neurons treated with BHBA for 1 h (Figure 3D). Similarly, p-CREB levels were much higher in the hippocampi of BHBA-treated mice than in those of control mice (Figure 3E). These results demonstrated that BHBA treatment triggered an elevation of intracellular Ca^2+^ and subsequently activated Ca^2+^/p-CaMKII/p-CREB signaling. Additionally, the binding of p-CREB to *Bdnf* promoters I and II was increased in murine hippocampal cell line HT22 after BHBA treatment for 1 h (Figure 3F). Increased binding of nuclear CREB-binding protein (CBP) was also observed in *Bdnf* promoter I. However, no changes were found in *Bdnf* promoter II in both BHBA-treated and untreated neurons (Figure 3G). Moreover, CBP levels were also significantly increased in the hippocampi of BHBA-treated mice and hippocampal neurons, repectively (Figures 3H,I). Thus, the BHBA-triggered elevation of intracellular calcium levels activated calcium-related signaling, which changed the binding of p-CREB/CBP to the *Bdnf* promoters to facilitate BDNF expression.

### Inactivation of Ca^2+^/CaMKII Inhibits BHBA-Induced BDNF

**FIGURE 4:**
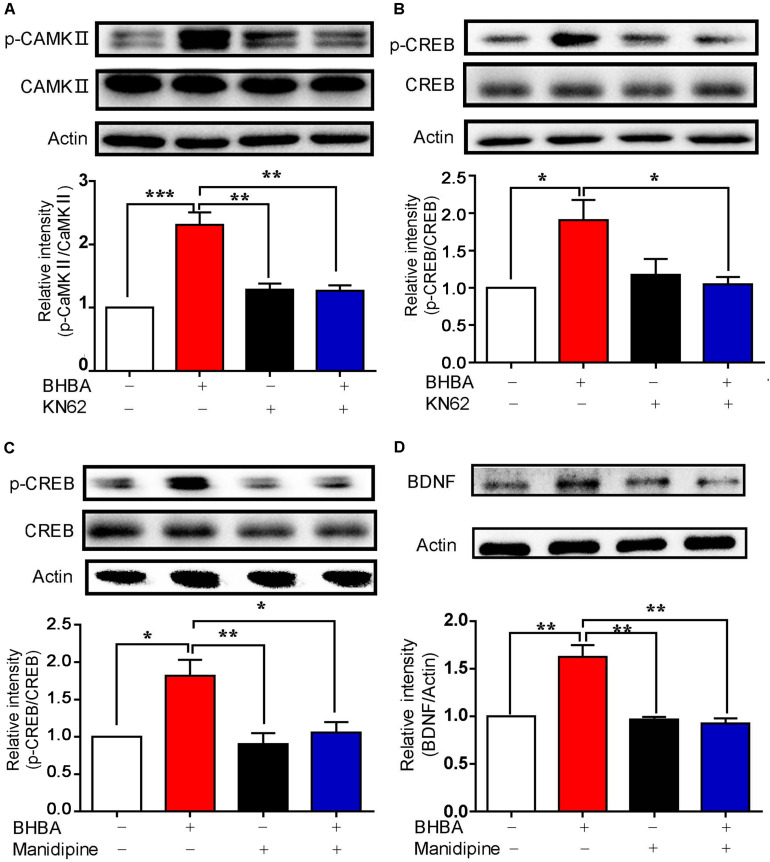
Inhibition of calcium signaling blocked the BHBA-induced increase of BDNF expression. **(A,B)** Western blot analysis of p-CaMKII (*n* = 3, *F*(3, 8) = 23.23) **(A)** and p-CREB (*n* = 3, *F*(3, 8) = 5.623) **(B)** levels in hippocampal neurons treated with 5 μM KN-62 for 10 min prior to treatment with 2 mM-BHBA treatment for another 30 min. **(C)** Western blot analysis of p-CREB levels in hippocampal neurons treated with 10 μM manidipine for 10 min or left untreated prior to treatment with 2 mM BHBA for 1 h (*n* = 4, *F*(3, 16) = 8.198). **(D)** Western blot analysis of BDNF expression in hippocampal neurons treated with 10 μM manidipine for 10 min or left untreated prior to treatment with 2 mM BHBA for 12 h (*n* = 4, *F*(3, 12) = 15.43). The data represent the mean ± SEM from three independent experiments. *p < 0.05, **p < 0.01, and ***p < 0.001.

To assess the role of calcium-related CaM/p-CaMKII signaling in BHBA-induced BDNF, hippocampal neurons were pre-treated with KN-62, a selective and specific inhibitor of CaM, before BHBA treatment. As expected, the BHBA-induced increase of p-CaMKII was reduced to the basal level by KN-62 (Figure 4A). At the same time, the elevation of p-CREB induced by BHBA was also decreased in KN-62-treated neurons, suggesting that the BHBA-induced rise of p-CREB levels depends on the activation of Ca^2+^/CaM/CaMKII (Figure 4B). Since Ca^2+^ influx through L-type VSCCs is one of the main sources of intracellular Ca2 +, hippocampal neurons were pre-treated with manidipine, a blocker of voltage-gated L-type VSCCs, to assess the role of L-type VSCCs in this process. The BHBA-induced increase of p-CREB levels was inhibited by manidipine, suggesting that L-type VSCCs are responsible for the elevation of p-CREB induced by BHBA (Figure 4C). A similar trend was observed for BDNF expression in hippocampal neurons pretreated with manidipine (Figure 4D). These findings supported the importance of L-type VSCCs and Ca^2+^/CaMKII in regulating the induction of BDNF expression by BHBA in hippocampal neurons.

### The BHBA-Induced Changes of H2AK119ub and H3K4me3 Depend on Different Ssignals

**FIGURE 5:**
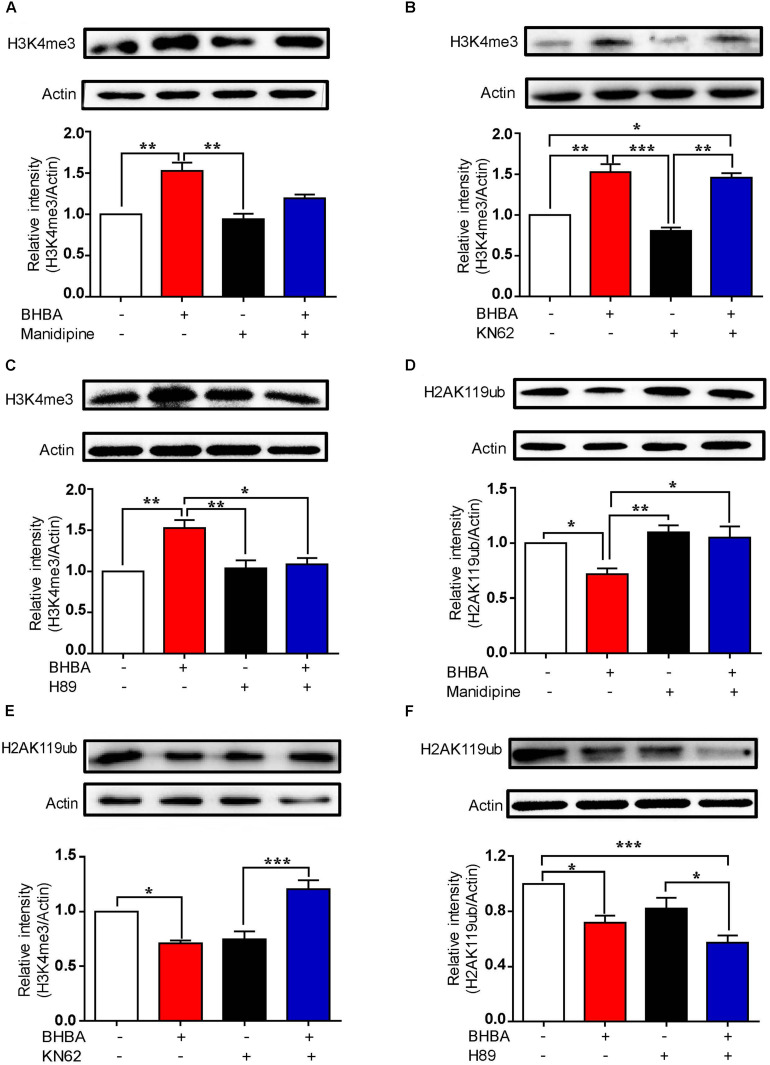
The BHBA-induced changes of H3K4me3 and H2AK119ub depend on different signaling pathways. **(A–C)** Western blot analysis of H3K4me3 levels in hippocampal neurons treated with 10 μM manidipine (*n* = 3 or 4, *F*(3, 10) = 11.27) **(A)**, 5 μM KN-62 (*n* = 3 or 4, *F*(3, 10) = 18.56) **(B)**, or 10 μM H89 (*n* = 3 or 4, *F*(3, 11) = 10.84) **(C)** for 10 min or left untreated prior to treatment with 2 mM BHBA for another 1 h. **(D, E)** Western blot analysis for H2AK119ub levels in hippocampal neurons treated with 10 μM manidipine (*n* = 3 or 4, *F*(3, 10) = 1.898) **(D)**, 5 μM KN-62 (*n* = 4, *F*(3, 12) = 16.95) **(E)**, or 10 μM H89 (*n* = 4, *F*(3, 12) = 10.61) **(F)** for 10 min or left untreated prior to treatment with 2 mM BHBA for another 1 h. The data represent the mean ± SEM from three independent experiments. *p < 0.05, **p < 0.01, and ***p < 0.001.

To further determine the roles of L-type VSCCs and Ca^2+^/CaM in regulating the BHBA-mediated increase of H3K4me3, hippocampal neurons were pre-treated with either manidipine or KN-62. The increase of H3K4me3 induced by BHBA was partially inhibited by manidipine, while KN-62 did not affect (Figures 5A,B), suggesting that the BHBA-induced upregulation of H3K4me3 is, at least in part, dependent on L-type VSCCs but not on CaM/p-CaMKII signaling. We previously found that BHBA also activated the cAMP/PKA signaling pathway in hippocampal neurons (Hu et al., 2018). To assess whether cAMP/PKA signaling plays a role in the BHBA-induced increase of H3K4me3, the hippocampal neurons were pre-treated with the PKA inhibitor H89 prior to BHBA stimulation. Notably, H89 completely blocked the promotion of H3K4me3 by BHBA, suggesting that cAMP/PKA signaling was responsible for the elevation of H3K4me3 levels following BHBA treatment (Figure 5C). Moreover, blocking the L-type VSCCs severely weakened the repression of H2AK119ub levels by BHBA, indicating that the BHBA-triggered activation of L-type VSCC-related calcium signaling resulted in the downregulation of H2AK119ub in hippocampal neurons (Figure 5D). The inhibition of BHBA on H2AK119ub level was also totally inhibited by KN-62 (Figure 5E). However, H89 did not change the inhibitory effect of BHBA on H2AK119ub at all, suggesting that the decrease of H2AK119ub levels was independent of cAMP/PKA signaling (Figure 5F). Collectively, these results demonstrated that the changes in H3K4me3 and H2AK119ub levels in hippocampal neurons following BHBA treatment are dependent on different signaling pathways. Overall, the results indicate that the decrease of H2AK119ub depends on L-type VSCCs and CaM/CaMKII signaling, and the increase of H3K4me3 depends on the activation of cAMP/PKA signaling.

## Discussion

It is well known that BHBA has a neuroprotective effect in the central nervous system, but the underlying mechanisms are the subject of ongoing research. As a ketone body synthesized primarily in the liver, BHBA can cross the blood–brain barrier to enter the brain and exert beneficial effects through multiple mechanisms (Newman and Verdin, 2017). In the present study, we showed that BHBA promoted the expression of Bdnf by regulating the histone modification on H2AK119 and H3K4. At the mean time, BHBA triggered an elevation of cytosolic Ca^2+^ in cultured hippocampal neurons. Our results are consistent with a previous study (Cheng et al., 2005), which showed that low concentrations of BHBA (0.02 g/l) can rapidly increase the intracellular Ca^2+^ levels in murine L929 fibroblasts, which was blocked by inhibitors of L-type VSCCs and EGTA. It is interesting that a fast slight increase of cytosolic Ca^2+^ was observed after BHBA stimulation, and then a very significant elevation Ca^2+^ level appeared. While whether these two peak were resulted form the intracellular Ca^2+^ release and the consequent extracellular Ca^2+^ influx, or there were corresponding to the HB induced Ca^2+^ signal and a subsequent neuronal firing are still need to be further investigated. Ca^2+^ signaling plays a pivotal role in regulating neuronal activity, and transient increases in cytoplasmic Ca^2+^ activate a variety of important calcium-dependent cellular events, including both the CaMK and PKA pathways (Bok et al., 2003; Hansen et al., 2003). We previously found that BHBA treatment could activate cAMP/PKA signaling, thereby elevating BDNF expression in hippocampal neurons (Hu et al., 2018). Here, we assessed the p-CaMKII level in hippocampal neurons treated with 2 mM BHBA or left untreated and found that BHBA effectively upregulated the p-CaMKII levels in hippocampal neurons. Although we didn’t observe the significant increase of p-CaMKII *in vivo* 12 h after BHBA administration (data not show), it might due to the highly dynamic property of CaMKII phosphorylation and dephosphorylation. CaMKII can phosphorylate CREB at serine 133, and once phosphorylated, CREB can recruit transcription coactivators such as CBP and its paralog p300, thereby activating the transcription of target genes. Moreover, the levels of p-CREB and BDNF in both BHBA-treated cultured hippocampal neurons and hippocampi of treated mice were much higher than those of the untreated control groups. At the same time, the binding of p-CREB and CBP to *Bdnf* promoters also increased, implying the activation of *Bdnf* transcription. These results suggested that the BHBA-triggered elevation of intracellular Ca^2+^ levels resulted in the activation of downstream signaling, which was beneficial to the function of the neurons. Transient elevation of intracellular Ca^2+^ can activate several transcription factors that facilitate neuronal function in addition to BDNF. Although we only detected the neuronal expression of BDNF in this study, it is believed that other Ca^2+^-dependent target genes are also affected by BHBA treatment, which could cooperatively facilitate neuronal activity. However, identifying those factors will require further analysis by RNA-seq or ChIP-seq in further studies. In addition to affecting L-type VSCCs in murine fibroblast L929 cells, BHBA was also reported to modulate *N*-type calcium channels and Cl^–^ channel in cultured neurons (Juge et al., 2010; Won et al., 2013). Moreover, it was found that BHBA could regulate the activity of sympathetic neurons via FFA3 (Won et al., 2013). Another study showed that ketone bodies, including acetoacetate and BHBA, inhibited glutamate transport by affecting the Cl^–^-dependence of VGLUT2 (Juge et al., 2010). These studies are in agreement with a pattern of BHBA modulating cellular functions by first affecting surface receptors. It has been reported that BHBA is a main endogenous ligand for two cell-surface receptors, GPR 109A and 41, both of which are Gi/o-protein-coupled receptors. We previously found that GPR109A but not GPR41 was expressed in the mouse hippocampal neuron line HT22. However, blocking Gi-type GPRs with PTX did not change the BHBA-induced increase of p-CREB, suggesting that GPR109A-coupled Gα/i was not responsible for BHBA-induced BDNF expression in neurons. It has been reported that activation of GPR109A is associated with an increase of intracellular Ca^2+^ levels through the Gβγ subunit/phospholipase Cβ (PLCβ)/inositol-1,4,5-trisphosphate (IP3) pathway in monocyte-derived cells (Offermanns and Schwaninger, 2015).

Our results demonstrated that blocking L-type VSCCs in hippocampal neurons also blocked the effects of BHBA on CaMKII/p-CREB activation, suggesting that the BHBA-induced increase of intracellular Ca^2+^ levels and subsequent activation of CaMKII/p-CREB/BDNF are mediated by L-type VSCCs. However, how BHBA modulates the activation of L-type VSCCs in neurons is still unclear and needs to be further investigated in the future.

It has been reported that BHBA can regulate BDNF transcription by affecting epigenetic modifications, such as increasing the occupancy of the transcriptional activation mark H3K27 acetylation and decreasing that of the transcriptional repression mark H3K27me3 at *Bdnf* promoters (Palomer et al., 2016; Hu et al., 2018). The transcriptional activation mark H3K4me3 was found to act in conjunction with H3K27me3 in a bivalent way in embryonic stem cells, giving them the ability to control gene expression rapidly in response to environmental changes (Bernstein et al., 2006). It has been shown that BHBA treatment decreased the occupancy of H3K27me3 at *Bdnf* promoters II and VI in the hippocampal neuron line HT22 (Hu et al., 2018). In this study, we found that BHBA increased global H3K4me3 levels. Moreover, the H3K4me3 occupancy at *Bdnf* promoters I, II, IV, and VI were also increased after BHBA treatment, indicating that H3K4me3 is involved in the increase of *Bdnf* expression in response to BHBA stimulations, possibly acting cooperatively with H3K27me3. H3K4me3 is a robust epigenetic mark that is involved in regulating synaptic plasticity as well as memory formation and consolidation. Webb et al. (2017) found that a recent contextual fear-conditioned memory elicited memory formation and elevated the global levels of H3K4me3 in the CA1 area. Collins et al. (2019) analyzed H3K4me3 ChIP-seq data from CA1 neurons and found broad H3K4me3 peaks at many learning- and memory-associated genes during memory formation. Moreover, H3K4 is specifically methylated by MLL and SETs, whereby MLL1 is widely expressed throughout the brain and plays crucial roles in synaptic activity and memory (Jakovcevski et al., 2015). The BHBA-induced elevation of WDR5, a core component of the MLL/SET complex, may explain why H3K4me3 levels increased after BHBA treatment since WDR5 is required for the tri-methylation of H3K4 (Wysocka et al., 2005). These results are consistent with BHBA’s neuroprotective role and indicate possible molecular mechanisms of its effects.

The histone site H2AK119 can also be mono-ubiquitinated by polycomb repressive complex 1 (PRC1) and Cullin4B-Ring E3 ligase complex (CRL4B) to repress target genes, and its role in tumorigenesis has been well characterized (Wang et al., 2004; Hu et al., 2012). However, the role of H2AK119ub in the central nervous system is still unclear. We found that the levels of the transcriptional repression mark H2AK119ub were reduced in cultured neurons and the hippocampi of treated mice, indicating that H2AK119ub might be involved in regulating neuronal functions. Other studies have shown that H2AK119ub plays a vital role in transcriptional repression by functionally coordinating with H3K27me3 (Muller and Verrijzer, 2009). In this study, we found that the binding of H2AK119ub to *Bdnf* promoters I, II, IV, and VI was reduced, indicating that H2AK119ub is involved in the BHBA-induced activation of *Bdnf* transcription. H3K27me3 is produced by the histone methyltransferase enhancer of Zeste homolog 2 (EZH2), the catalytic subunit of PRC2, and it is typically added to nucleosomes across upstream promoters and coding regions of PRC2-targeted genes to repress them (Xu et al., 2012). In addition, H3K27me3 was also found to interact with PRC1, while the mono-ubiquitination of H2AK119 by PRC1, in turn, promotes the tri-methylation of H3K27 on H2Aub nucleosomes (Kalb et al., 2014). The fact that H2AK119ub can functionally interact with H3K27me3 also explains the reduction of H2K119ub binding at *Bdnf* promoters after BHBA treatment. Notably, the occupancy of H2AK119ub at *Bdnf* promoters I, II, IV, and VI was decreased, while only the occupancy of H3K27me at *Bdnf* II and VI was found to be reduced by BHBA in our previous study (Hu et al., 2018). CUL4B is a vital component responsible for H2AK119 ubiquitination, acting as a scaffold protein that assembles multiple E3 complexes, including CRL4B via the linker protein DDB1 (Nakagawa and Xiong, 2011a). It has been shown that CUL4B binds to the promoters or enhancer regions of targeted genes, together with EZH2, to silence their transcription by catalyzing H2AK119 mono-ubiquitination and H3K27 tri-methylation (Hu et al., 2012). Although PRC1 and PRC2 usually co-localize at target sites in the chromatin, they rely on different mechanisms. In the “hierarchical” model, the initial event is the binding of PRC2 to chromatin. PRC2 then catalyzes H3K27 tri-methylation, which subsequently recruits PRC1 by interaction with the chromo box (CBX)-containing protein, leading to H2AK119 mono-ubiquitination. In the “alternative” model, PRC1 is recruited to chromatin and then mono-ubiquitinates H2AK119 independently of PRC2 activity (Blackledge et al., 2015). This means that H2AK119ub and H3K27me3 are not always introduced at the same target sites, although most of the time, they work together to inhibit gene transcription. Furthermore, CUL4B can regulate the expression of neuronal genes such as SLC6A12, SYN1, and SNAP2, by targeting the methyltransferase component of H3K4me3, WDR5, for ubiquitination and degradation (Nakagawa and Xiong, 2011b). Together with the discussed literature, our results indicate the possibility that H2AK119ub plays a role in regulating neuronal function by acting cooperatively with H3K4me3 and H3K27me3, allowing neurons to rapidly respond to environmental changes. However, direct evidence for this hypothesis is still lacking. In addition, another study showed that the H2AK119 E3 ubiquitin ligase 2A-HUB can be recruited by N-CoR/HDAC1/3 to form a complex that regulates chromatin remodeling and gene transcription by promoting H2A monoubiquitination (Zhou et al., 2008). Zhu et al. (2007) found that H2A de-ubiquitinase (2A-DUB) interacts with the histone acetyltransferase (HAT) p300/CBP-associated factor (p/CAF) to form an enzymatic complex, which could help HATs provide “optimized” substrates for 2A-DUB to remove the ubiquitin from H2A. As an inhibitor of class-I HADCs, BHBA might affect H2AK119ub levels by changing the interaction of 2A-HUB and N-CoR/HDAC1/3 at the *Bdnf* promoters, or by promoting the formation of p/CAF complexes with 2A-DUB to remove the ubiquitin from H2AK119.

Finally, we found that the BHBA-induced upregulation of H3K4me3 and downregulation of H2AK119ub is dependent on L-type VSCCs by using manidipine to block the impact of BHBA on H3K4me3 and H2AK119ub in neurons. However, we are not aware of reports of any direct evidence demonstrating a relation between H3K4 tri-methylation or H2AK119 ubiquitination with calcium signaling. One study showed that the abundance of the CUL4B-PRC2 complex increases in response to high-glucose stimulus and affects intracellular Ca^2+^ by decreasing Cav1.2 expression (Li et al., 2017). However, in our study, the effect of BHBA on intracellular Ca^2+^ was fast, suggesting that BHBA might directly affect the opening and closing of L-type VSCCs. Moreover, the changes of H3K4me3 and H2AK119ub were found to be downstream of Ca^2+^ signaling. However, it is possible that the BHBA-induced changes of H2AK119ub, in turn, can further influence the Ca^2+^ concentration by affecting the expression of calcium channels such as Cav1.2.

**FIGURE 6:**
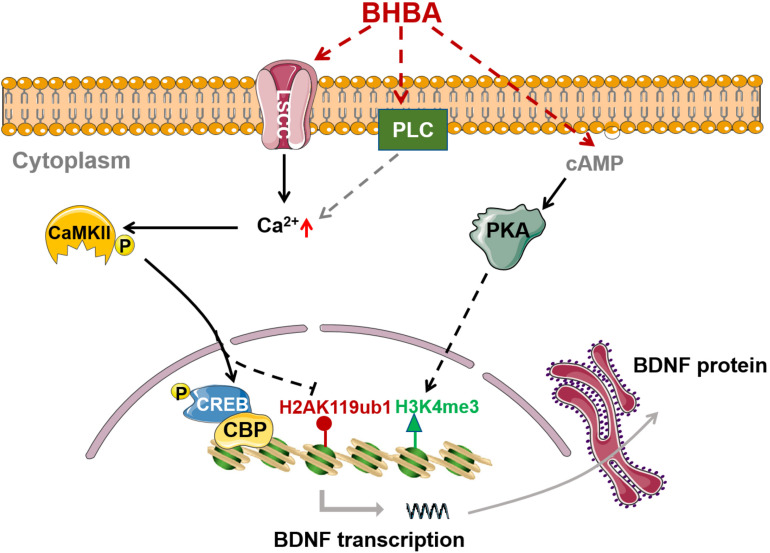
BHBA-induced activation of intracellular signaling and chromatin remodeling in Bdnf promoters in hippocampal neurons. BHBA induces increase of transcriptional mark H3K4me3 as well as decrease of transcriptional mark H2AK119ub occupied in bdnf promoters in hippocampal neurons. Moreover, BHBA-induced changes of H2AK119ub is dependent on the activation of L-type calcium channel and the elevation of intracellular, which subsequently activates Ca^2+^/CaMKII/p-CREB signaling pathway, and promotes the occupation of p-CREB and CBP in bdnf promoters, thereby enhances BDNF expression. However, BHBA-increased H3K4me3 is dependent on cAMP/PKA activation.

In summary, our study revealed that BHAB stimulation increased histone H3K4me3 and decreased H2AK119ub in hippocampal neurons both *in vitro* and *in vivo*. The occupancy of H3K4me3 and H2AK119ub at *Bdnf* promoters was also correspondingly enhanced and decreased, which contributed to the BHBA-induced expression of BDNF. Moreover, BHBA triggered an increase of intracellular Ca^2+^ via L-type VSCCs, which in turn activated the Ca^2+^/CaMKII/p-CREB signaling pathway and subsequently induced BDNF expression in primary hippocampal neurons. Furthermore, the BHBA-induced decrease of H2AK119ub was dependent on L-type VSCCs, while the increase of H3K4me3 mainly resulted from BHBA-activated cAMP/PKA signaling (Figure 6). Taken together, our findings further enrich the knowledge on the molecular mechanisms by which BHBA induces *Bdnf* expression. We revealed that BHBA also regulates the levels of H2AK119ub and H3K4me3 in addition to the previously reported H3K27me3/ac. These findings broaden the roles of BHBA in modulating the histone code and provides new perspectives for understanding the beneficial effects of BHBA on the CNS.

## Data Availability Statement

The raw data supporting the conclusions of this article will be made available by the authors, without undue reservation.

## Ethics Statement

The animal study was reviewed and approved by the Research Ethics Committee of Xi’an Jiaotong University.

## Author Contributions

XL, YZ, and EH designed the study. EH, HD, and SS carried out the experiments, the data analysis and contributed to preparing the manuscript. All authors discussed the results and contributed to the final manuscript.

## Conflict of Interest

The authors declare that the research was conducted in the absence of any commercial or financial relationships that could be construed as a potential conflict of interest.
